# Electromembrane Extraction Provides Unprecedented Selectivity for Drugs in Cell Culture Media Used in Organoid and Organ-on-Chip Systems

**DOI:** 10.1021/acs.analchem.4c04994

**Published:** 2025-02-25

**Authors:** Stian Kogler, Fro̷ydis Sved Skottvoll, Helena Hrušková, Frode Rise, Aleksandra Aizenshtadt, Stefan Krauss, Hanne Ro̷berg-Larsen, Frederik André Hansen, Steven Ray Wilson

**Affiliations:** 1 Section for Chemical Life Sciences, Department of Chemistry, Faculty of Mathematics and Natural Sciences, University of Oslo, Oslo 0371, Norway; 2 Hybrid Technology Hub - Centre of Excellence, Institute of Basic Medical Sciences, Faculty of Medicine, University of Oslo, Oslo 0315, Norway; 3 Department of Smart Sensors and Microsystems, SINTEF Digital, Oslo 0373, Norway; 4 Section for Catalysis and Organic Chemistry, Department of Chemistry, Faculty of Mathematics and Natural Sciences, University of Oslo, Oslo 0371, Norway; 5 Department of Pharmacy, Faculty of Mathematics and Natural Sciences, University of Oslo, Oslo 0371, Norway

## Abstract

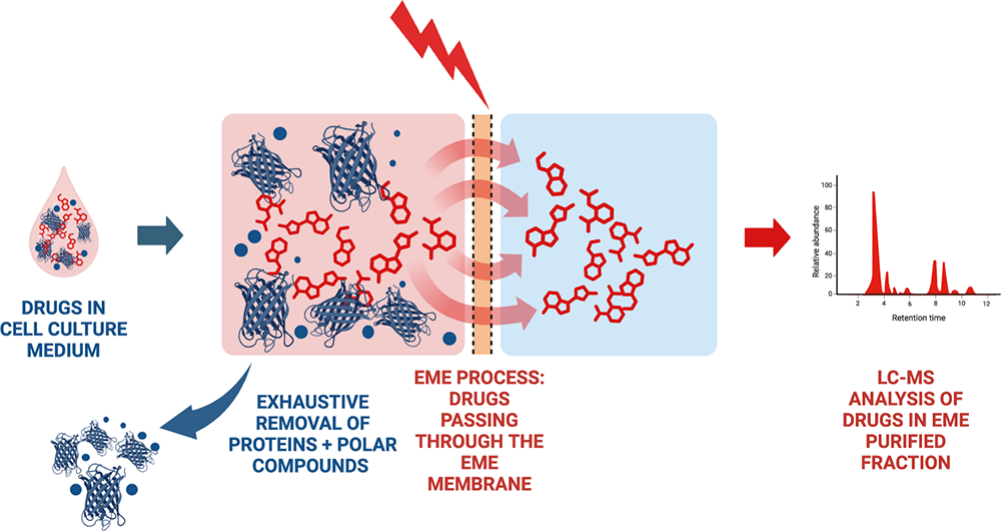

The use of organoids and organ-on-chip technologies as nonanimal methodologies in drug discovery and personalized medicine is rapidly expanding. However, the complexity and small volumes of organoid culture samples present significant analytical challenges, e.g., in drug analysis using liquid chromatography–mass spectrometry (LC–MS). Essentially an electrophoresis across an oil membrane, electromembrane extraction (EME) offers a promising approach for measuring drugs, as it is, for example, compatible with small samples such as organoid and organ-on-chip formats. Given the potential of the technology, there is a need to assess the extraction purity of EME extracts to ensure EME’s compatibility with high-throughput, downstream analysis. This study evaluates the effectiveness of EME for sample cleanup in various common cell culture media used for organoids and organs-on-chips. The media were spiked with 90 small-molecule drugs. Using gel electrophoresis (sodium dodecyl sulfate polyacrylamide gel electrophoresis), high-resolution nuclear magnetic resonance spectroscopy, and LC–MS, we demonstrate that EME provides exhaustive removal of unwanted medium components (proteins, polar molecules, and apolar/neutral molecules) while selectively extracting the spiked small-molecule drugs. The approach was demonstrated with human stem-cell-derived liver organoids, allowing simple detection and monitoring of telltale cytochrome P450 metabolism. Taken together, our observations highlight an unprecedented ability of EME to provide sample cleanup for drug analysis in matrixes compatible with organoids and organ-on-chip technology.

## Introduction

Societal incentives to reduce the use of animal models in research are driven by ethical concerns and animals’ limited ability to predict human physiology. This shift is further supported by legislative changes such as the EU’s REACH regulation and the U.S. EPA’s directive to reduce animal testing by 2035. As a result, nonanimal methodologies (NAMs) have evolved, including organoids and organ-on-chip (OoC) technology,^1,2^ which provide a miniaturized recapitulation of human physiology. Derived from sources like patient-induced pluripotent stem cells, patient-derived progenitor cells, or mature functional cells, these organ models can imitate aspects of human organ functions. Placed in a controlled microphysiological setting, organoids and OoC technology can simulate specific conditions of human organ systems,^3^ offering deeper insights into organ physiology, disease mechanisms, drug discovery, and personalized medicine. Consequently, these technologies are becoming major focus areas for academia, pharmaceutical companies, and contract research organizations (CROs).

As organoid/OoC markets are rapidly growing, the need for robust and high-throughput analytical tools for chemical analysis, e.g., drug metabolism measurements, becomes increasingly important. Today’s drug analysis platforms are mostly based on liquid chromatography coupled to mass spectrometry (LC–MS), which allows unprecedented specificity, i.e., allowing highly similar molecules to be distinguished and accurately measured.^4^ However, even with the vast separation powers and sensitivity of today’s LC–MS systems, extensive sample preparation of biosamples can be called for to avoid system clogging and perturbed measurements. Preparation steps can include protein precipitations, liquid–liquid extractions, and solid-phase extractions. In addition, such sample preparation steps may not be optimal for smaller samples, e.g., microliter/nanoliter amounts of organoid culture medium samples extracted from an OoC system. As a result, the progress and utility of organoids and OoCs have bottlenecks in chemical analysis.

We previously developed and evaluated several sample preparation approaches for organoid/OoC samples. For drug/drug metabolite analysis, we have recently developed an LC–MS system with online sample preparation, where salts and proteins are removed using a self-cleaning solid-phase extraction (SPE) system.^5^ In parallel, we are studying electromembrane extraction (EME)-based approaches,^6^ as we find EME to be potentially suited for direct coupling/online monitoring of organoid/OoC systems and minute samples,^7^ i.e., hosting organoids within a chip and selectively extracting and directing drugs/drug metabolites to LC–MS in a fully automated fashion.

EME is a three-phase microextraction technique applied to isolating charged compounds from a sample. This process involves the transfer of these compounds across a liquid membrane that is retained within the pores of a polymeric membrane, commonly termed a supported liquid membrane (SLM), and subsequently into a clean aqueous acceptor solution. The migration across the SLM is facilitated by an electric field, which, in combination with the few microliters of solvent used in the SLM, dictates the selectivity of the system. EME has been realized in different technical formats, including on-chip^8^ and 96-well formats,^6^ and was recently commercialized in a format based on conductive vials.^9^

To be a candidate for analysis of organoid/OoC samples beyond proof-of-concept studies (i.e., applied, robust, high-throughput, and automated analysis), a documentation of the sample preparation traits of EME is called for. Few, if any, studies have focused beyond selective reaction monitoring (SRM) transitions, e.g., focusing on the analytes’ yield rather than the fate of the unwanted matrix. While NMR has previously been used to assess metabolic profiles in organoid samples,^10^ no studies have utilized NMR to evaluate sample cleanup. Moreover, very few papers have focused on the applicability of EME with organoid culture medium,^6,7^ which features a plethora of potential interferents, namely, salts, proteins, and small-molecule nutrients (amino acids, sugars, etc.).

Therefore, the aim of this study was to assess the sample cleanup effect of EME, using SDS-PAGE for analyzing the removal/extraction of proteins, 800 MHz NMR spectroscopy for profiling small-molecule nutrients, and LC–MS for determining the recovery and matrix effects on 90 drugs spiked to various culture media and benchmarked to more familiar matrixes and preparations, namely, plasma and various protein precipitation procedures used in clinical applications. The approach was demonstrated with the analysis of liver organoid drug metabolism. In summary, our investigations show a virtually exhaustive cleanup when applying EME, while small-molecule drugs are selectively extracted.

## Experimental Section

### Medium and Drug Mixture

The base medium for all medium formulations used in this study was William’s E medium, Glutamax supplemented (catalog number: 32551020) and purchased from Thermo Fisher Scientific. This medium was used as is as medium 1 (hereafter referred to as WE-medium). Media 2 and 3 were further supplemented with 1% nonessential amino acid solution, 0.1% insulin–transferrin–selenium and 0.1% penicillin–streptomycin solution (all purchased from Thermo Fisher Scientific). Additionally, medium 2 (hereafter termed BSA-medium) was supplemented with bovine serum albumin (BSA) to a 1% solution (w/v, VWR), while medium 3 (hereafter termed FBS-medium) was supplemented with 1% FBS (v/v, Thermo Fisher Scientific). A sample of human serum (pooled from six donors) was also used and was purchased from the blood bank of Oslo University Hospital (Norway).

A mixture of 90 drugs was prepared according to Zhou et al.^11^ A list of all drugs, including mass, p*K*_a_, and log *P*, is shown in Supporting Information (SI) 1. The concentration of each compound in the spiking mixture was 5 μg/mL. The drug mixture was spiked and thoroughly mixed with the matrixes 30 min before the extraction procedure to a final concentration of 100 ng/mL of each compound.

### EME and LC–MS

Aliquots of 120 μL of each spiked sample were transferred into 200 μL conductive vials from the ETN-12 EME apparatus from Extraction Technologies Norway (Oslo, Norway). Aliquots of 9 μL of 2-nitrophenyl octyl ether (NPOE) (Sigma-Aldrich, St. Louis, MO, USA) were loaded onto the support membrane to serve as the liquid membrane. Aliquots of 120 μL of 100 mM formic acid (pH 2.4) were used as acceptor solution and were filled into the second conductive vial. The extraction was run at 50 V for 15 min. LC–MS analysis of extracts/acceptor solutions was performed as previously described by Zhou et al.^11^

### Protein Precipitation

Protein precipitation (PPT) was performed on samples of WE-medium, BSA-medium, and FBS-medium and human plasma according to three standard procedures with methanol^12^ (PPT1), a mixture of acetonitrile/methanol (85/15 v/v)^13^ (PPT2), and acetonitrile^14^ (PPT3), which are in routine use in clinical settings. All procedures were scaled to 100 μL of the sample. After the removal of the supernatant from the precipitate, the samples were evaporated at 45 °C to dryness, followed by resuspension in 100 μL of water. For procedure 2, 375 μL of the supernatant was collected.

### Gel Electrophoresis (SDS-PAGE)

Samples of BSA-medium and FBS-medium and plasma were diluted 1:2 with water before mixing with loading buffer to avoid overdosing. WE-medium (containing no proteins) and all of the other samples (EME extracts and PPT-treated samples) were used undiluted. Samples were then prepared and run according to the procedure by Olsen et al.^15^ A protein ladder was run with each SDS-PAGE experiment.

### NMR Spectroscopy

For NMR analysis, 200 μL of undiluted donor or pooled acceptor solution (*n* = 3) was transferred together with 50 μL of D_2_O and 250 μL of H_2_O to a 5 mm O.D. Wilmad economy NMR tube. As control, an NMR tube with 500 μL of H_2_O/D_2_O was prepared by mixing 450 μL of water with 50 μL of D_2_O. Additionally, an NMR tube with H_2_O/D_2_O/100 mM FA (250/50/200 μL) was prepared. ^1^H NMR one-dimensional data was acquired on a Bruker Avance Neo 800 instrument (800.03 MHz) (Bruker, Fällanden, Switzerland) equipped with a 5 mm TCI (^1^H^15^N^13^C) cryo probe using the software TopSpin 4.3.0 for acquisition and TopSpin 4.2.0 for processing. Solvent (water) suppression was accomplished with presaturation using the pulse program zgpr with pldb9 set to +40.08. The 90° pulse width was 8.0 μs at pldb1 −9.84. A relaxation delay (D1) of 2 s was used. An acquisition time of 4.46 s with SW 19.38 ppm and TD 131072 were used. Four dummy scans and 16 acquisition scans were employed on each sample. The suppression point (O1) was individually optimized for each sample.

### Liver Organoid Culture and Drug Exposure

Liver organoids were generated from human induced pluripotent stem cells (WTC-11, Coriell Institute for Medical Research) according to a previously published protocol.^3^ Generated liver organoids (25 organoids per replicate) were incubated with diltiazem at a concentration of 1 μM in William’s E medium, supplemented with 0.1% (v/v) insulin–transferrin–selenium, 1% (v/v) MEM nonessential amino acid solution, 1% (v/v) Glutamax, and 0.1% (v/v) BSA (total volume of 600 μL; all reagents were purchased from Thermo Fisher Scientific) in a humidified 37 °C, 5% CO_2_ incubator. Media were sampled after 2, 4, 6, and 24 h of incubation and stored at −80 °C prior to analysis. To control nonenzymatic drug metabolism, samples of media without organoids were collected at the same time points.

### Software and Calculations

Log *P*, pI, and p*K*_a_ values (found in Table 1) were retrieved from the Chemicalize software (Chemaxon, Budapest, Hungary).

**Table 1:** Overview of CCM Small Organic Molecules and Log *P*, p*K*_a_, and pI (Where Applicable)

**amino acids**	log *P*	**p***K*_**a**_**and pI values**
l-alanine	–2.841	pI 5.98
l-arginine	–3.156	pI 10.77
l-asparagine	–4.288	pI 5.23
l-aspartic acid	–3.504	pI 3.21
l-cysteine	–2.795	pI 4.27
l-cystine	–5.898	pI 5.24
l-glutamic acid	–3.249	pI 3.49
l-glutamine	–4.001	pI 5.74
glycine	–3.409	pI 5.77
l-histidine	–3.616	pI 8.02
hydroxy-l-proline	–3.716	pI 5.63
l-isoleucine	–1.508	pI 6.19
l-leucine	–1.586	pI 6.15
l-lysine	–3.215	pI 9.82
l-methionine	–2.189	pI 6.02
l-phenylalanine	–2.189	pI 6.02
l-proline	–2.569	pI 6.14
l-serine	–3.888	pI 5.48
l-threonine	–3.471	pI 5.6
L-tryptophan	–1.085	pI 5.97
l-tyrosine	–1.489	pI 5.51
l-valine	–1.953	pI 6.16

**vitamins**	**log *P***	p*K*_a_/pI
ascorbic acid		strongest acidic p*K*_a_: 4.03
		pI 1.12
d-biotin	0.319	strongest acidic p*K*_a_: 4.4
		strongest alkaline p*K*_a_: −1.86
		pI 1.27
calciferol	7.051	strongest alkaline p*K*_a_: −1.32
choline chloride	4.662	strongest acidic p*K*_a_: 13.97
folic acid	–0.739	Strongest acidic p*K*_a_: 4.02
		strongest alkaline p*K*_a_: 2.82
		pI 3.37
myoinositol	–3.782	strongest acidic p*K*_a_: 12.2
menadione (sodium bisulfite)	1.89	N/A
niacinamide	–0.394	strongest acidic p*K*_a_: 13.39
		strongest alkaline p*K*_a_: 3.63
		pI 8.51
*p*-aminobenzoic acid	0.802	strongest acidic p*K*_a_: 4.77
		strongest alkaline p*K*_a_: 2.69
		pI 3.72
d-pantothenic acid	1.539	strongest acidic p*K*_a_: 4.36
		strongest alkaline p*K*_a_: 2.79
		pI 3.57
pyridoxal	0.179	strongest acidic p*K*_a_: 8.17
		strongest alkaline p*K*_a_: 4.31
		pI 6.24
pyridoxine	–0.951	strongest acidic p*K*_a_: 9
		strongest alkaline p*K*_a_: 5.19
		pI 7.09
retinol	4.694	N/A
riboflavin	–0.917	strongest acidic p*K*_a_: 5.97
		strongest alkaline p*K*_a_: 0.78
		pI 3.36
thiamine	–3.097	strongest acidic p*K*_a_: 15.5
		strongest alkaline p*K*_a_: 5.54
vitamin B12	–2.695	strongest acidic p*K*_a_: 1.42
		strongest alkaline p*K*_a_: 8.94
		pI 8.41

**others**	**log *P***	**p*K*_a_/pI**
d-glucose	–3.568	N/A
glutathione (reduced)	–4.92	strongest acidic p*K*_a_: 1.94
		strongest alkaline p*K*_a_: 9.31
		pI 3.07
methyl linoleate	6.568	N/A
phenol red	4.109	strongest acidic p*K*_a_: 9.16
pyruvic acid	0.066	strongest acidic p*K*_a_: 2.93

## Results and Discussion

EME (conductive vial format) was performed on a mixture of 90 alkaline drugs (p*K*_a_ range: 0.5–12.2), which spanned a fairly wide hydrophobic range (log *P* −2.0 to +8.1). The EME conditions were fairly generic for this approach, namely, applying an electric potential of 50 V and an NPOE oil for extraction between the donor and acceptor solution. NPOE has been shown to provide recovery particularly for alkaline drugs with a log *P* range of 2–6 (for calibration to current drug development: the average calculated log *P* for recently approved small-molecule drugs is 3.1^16^). The samples investigated were several types of cell culture media (CCM) that are commonly used for organ models. These samples are highly complex, both regarding small and large molecules (amino acids, sugars, proteins, etc.).^5^

### SDS-PAGE Reveals the Absence of Protein in the Extracts

**Figure 1:**
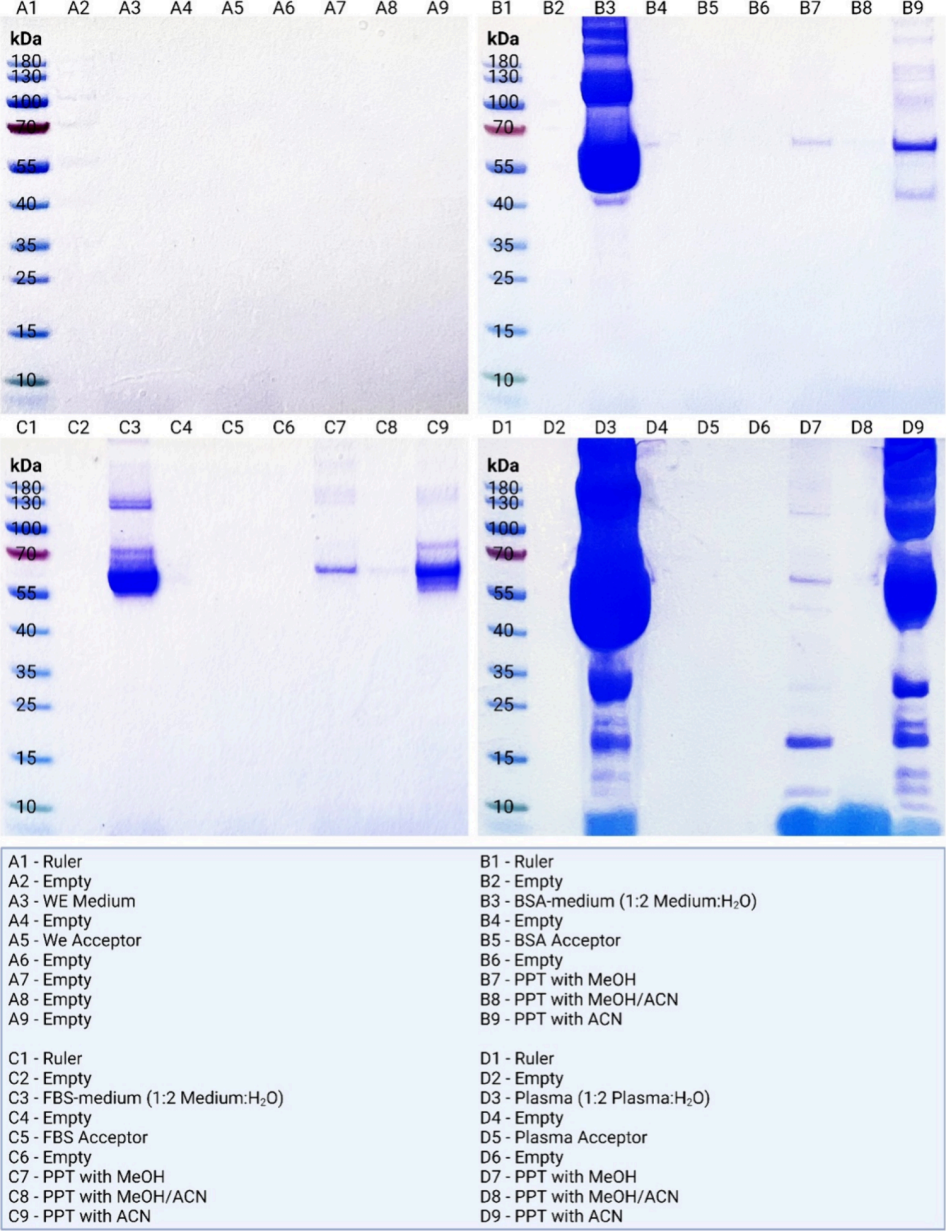
SDS-PAGE of medium samples, EME extracts, and medium after three different PPT methods. The gels reveal the complete removal of protein by EME, while commonly used PPT methods show the leftover protein in the samples.

In this part, we focused on the evaluation of the purification efficiency of EME. The ability of this technique to isolate samples from proteins was studied by SDS-PAGE and compared to three routine PPT protocols (Figure 1). Unedited pictures of all gels are shown in SI 2 and 3.

The control sample containing no proteins provided clean lanes for the donor and EME-acceptor solution, as expected (Figure 1, lanes A3 and A5). Investigated donor samples were rich in protein content, as visible in Figure 1, lanes B3, C3, and D3. The pretreatment of these samples by EME provided exhaustive elimination of the proteins as no protein bands were detected (estimated LOD = 30–300 ng of protein; see SI 4) in the acceptor solutions (Figure 1, lanes B5, C5, and D5).

Furthermore, EME was also compared with the results of three commonly applied standard PPTs. Residues of the large proteins were found in the majority of the PPT-treated samples as visible in the corresponding gel lanes (Figure 1, lanes B7, B8, B9, C7, C8, C9, D7, D8, and D9). PPT2 seemed to have superior purification properties over the other PPTs and was comparable to EME (Figure 1, lanes B8, C8, and D8). This protocol is specific by employing the combination of two different solvents, potentially improving the purification feasibility. On the other hand, PPT3 (Figure 1, lanes B9, C9, and D9) lacked effectiveness as large amounts of proteins were found in the treated samples (especially for the plasma samples). Difficulties of this particular procedure could be linked to the addition of a relatively low volume of organic solvent (lower than that in other PPT procedures), resulting in higher operational demands on sample handling during the PPT3 procedure. All three protocols include drying and redissolving the sample in water or large dilutions to make treated samples compatible with reversed-phase LC. Drying and resolving in water are very time-consuming steps, while dilution of the sample can lead to sensitivity issues for low-abundance analytes. In comparison, the EME in this study was run for 15 min in total and has the possibility to enrich samples. In conclusion, classic SDS-PAGE shows that EME provides an extremely efficient and fast organic solvent-free cleanup regarding protein removal from CCM samples and superior purification efficiency compared with two out of three commonly used PPTs with a high degree of selectivity.

### NMR Reveals EME Removal of Small Molecules from CCM

**Figure 2:**
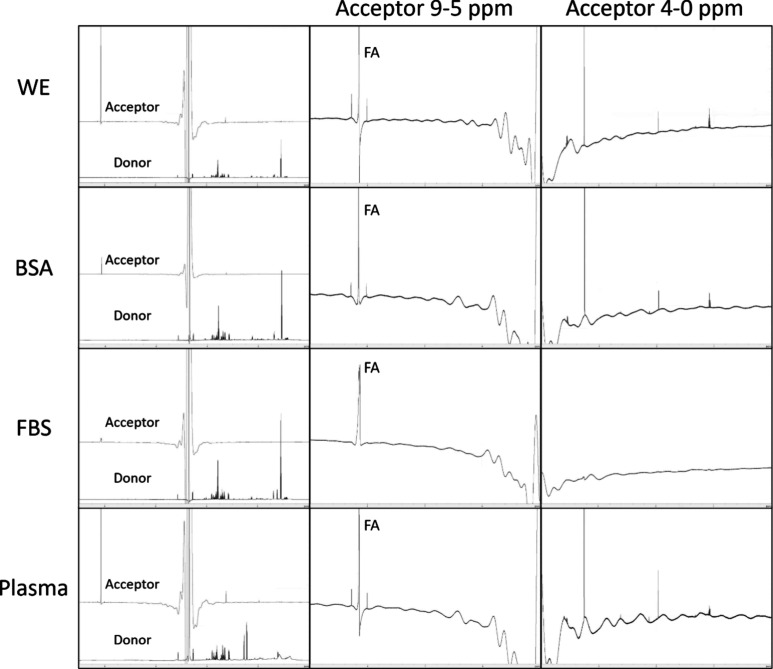
NMR of untreated donor and arising acceptor solutions revealed close to complete removal of small molecules through the EME process. Left: overlay of NMR spectra of the donor and acceptor. See SI 1–4 for large images. Middle and right: zoom-in of acceptor solutions with excluded water peak. FA is part of the solvent that comprises the acceptor solution.

CCM will also contain a host of inorganic salts, as well as a number of organic molecules, e.g., amino acids and vitamins. The organic molecules will be present in a relatively wide range of concentrations, up to several mg/mL (glucose). In addition, the chemical properties vary substantially, e.g., in hydrophobicity (see Table 1). Considering that the EME conditions would be particularly tuned for alkaline molecules with a log *P* range of 2 to 6, we hypothesized that most of the polar amino acids, etc., would not reach the acceptor solution. With high-resolution NMR spectroscopy, we were able to profile the contents of the donor and acceptor solutions (Figure 2). Indeed, the donor solutions (i.e., CCM and plasma) showed abundant and complex NMR spectra in the expected shift areas of the amino acids, glucose, etc. (see also SI 5–8). However, the acceptor solutions were remarkably clean of small molecules, with the exceptions of the formic acid peak (deliberately added to the acceptor solution) and a few unidentified trace singlets. We note that several of the CCM substances have log *P* values that are within the “sweet spot” of the EME conditions (Table 1). However, these molecules do not have an alkaline group and struggle to migrate with the applied electric field, explaining the absence in the NMR spectra of the acceptor solutions. In conclusion, NMR spectroscopy could reveal virtually exhaustive removal of the small molecules present in common CCM solutions.

### Recovery of Compounds (LC–MS)

**Figure 3:**
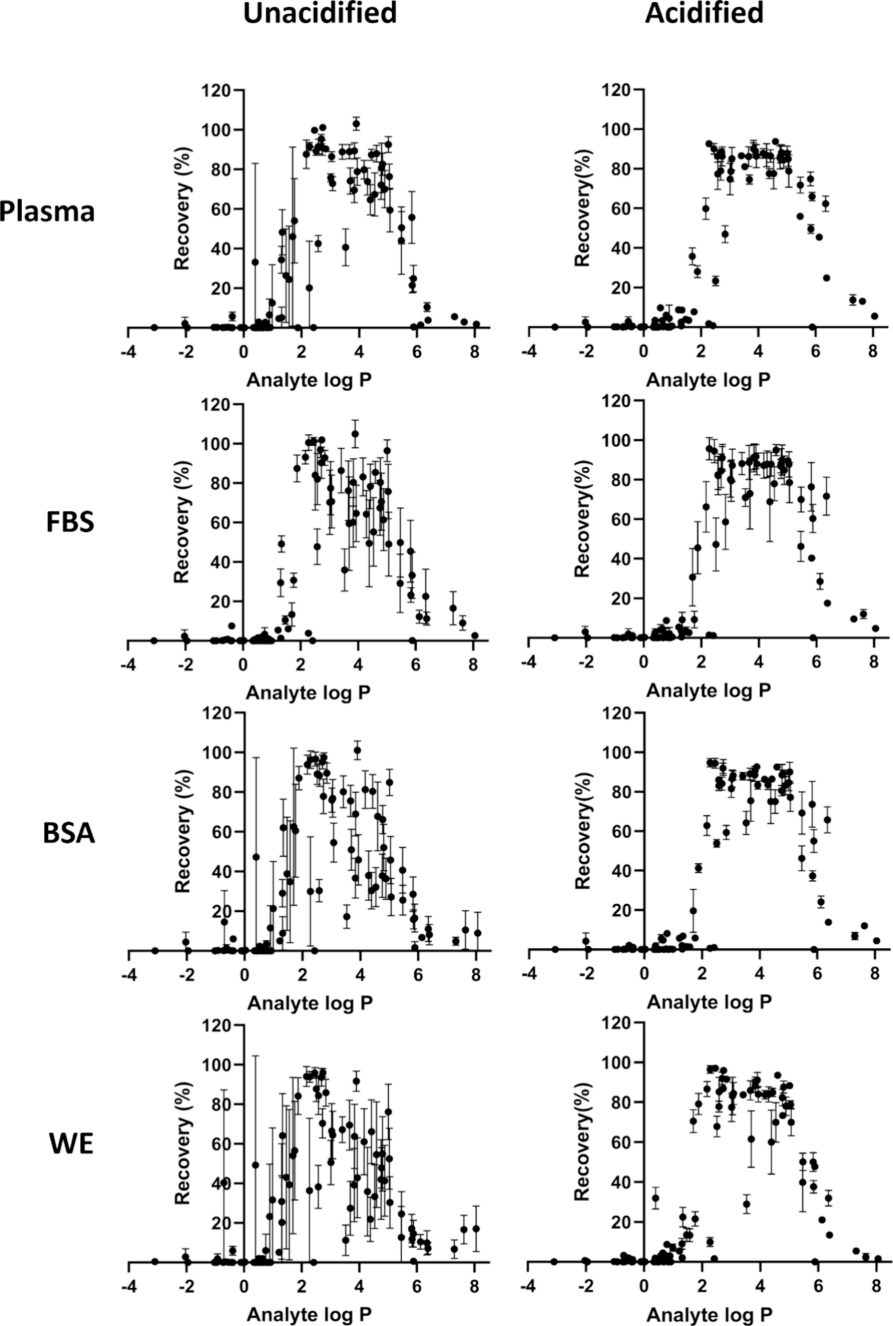
Plots of all 90 compounds’ recovery plotted against the analytes’ log *P* values in both acidified and unacidified donor solutions.

After establishing a highly efficient cleanup regarding unwanted compounds from CCM, we focused on the extraction traits of the analytes, i.e., 90 alkaline drugs that were spiked to the various matrixes. Plasma samples have been substantially studied regarding EME optimization using NPOE.^17^ For conventional EME conditions (donor solution acidified, arguably reducing the %protein binding,^18^ a plateau of highly recovered drugs (80–100%) spanned approximately 3–5 log *P*, with similar patterns for all samples (Figure 3).

**Figure 4:**
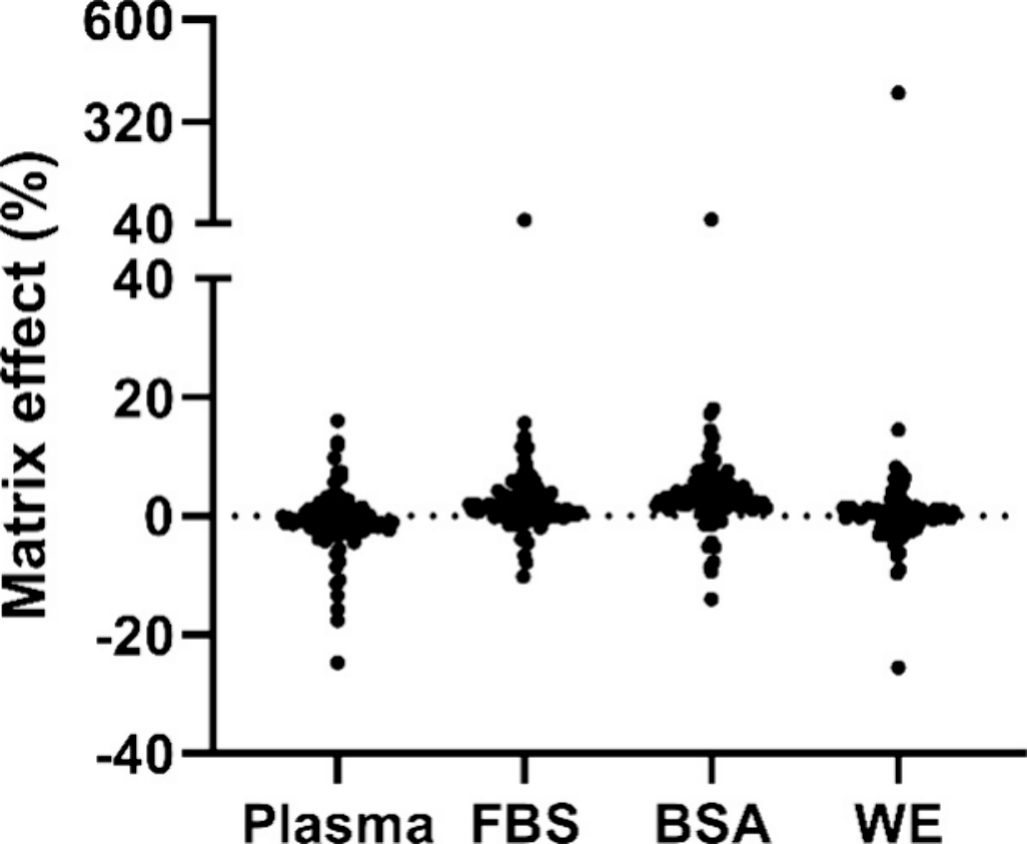
Matrix effect for all 90 compounds for unacidified donor solutions. Values are normalized to a clean acceptor solution spiked with the compounds with negative and positive values representing ion suppression and ion enhancement, respectively. Most compounds in all matrixes are close to 0% (no matrix effect), showing selective extraction of alkaline drugs. Differences between the four matrixes were not statistically significant (*p* = 0.37).

With a simplified EME procedure (i.e., not adding acid to the donor solutions), drugs could still be recovered, but with more variance (Figure 3). It could be speculated that the acidification contributes to reduction of variance due to weakening drug–protein interactions. We have observed that variances in recoveries with CCM and EME can be corrected for using internal standards,^6^ but the results here suggest clear performance benefits of an acid addition to the donor solutions. Considering the substantial cleanup of the unwanted matrix components, it was unsurprising that matrix effects in LC–MS analysis were unsubstantial for the samples (see Figure 4).

**Figure 5:**
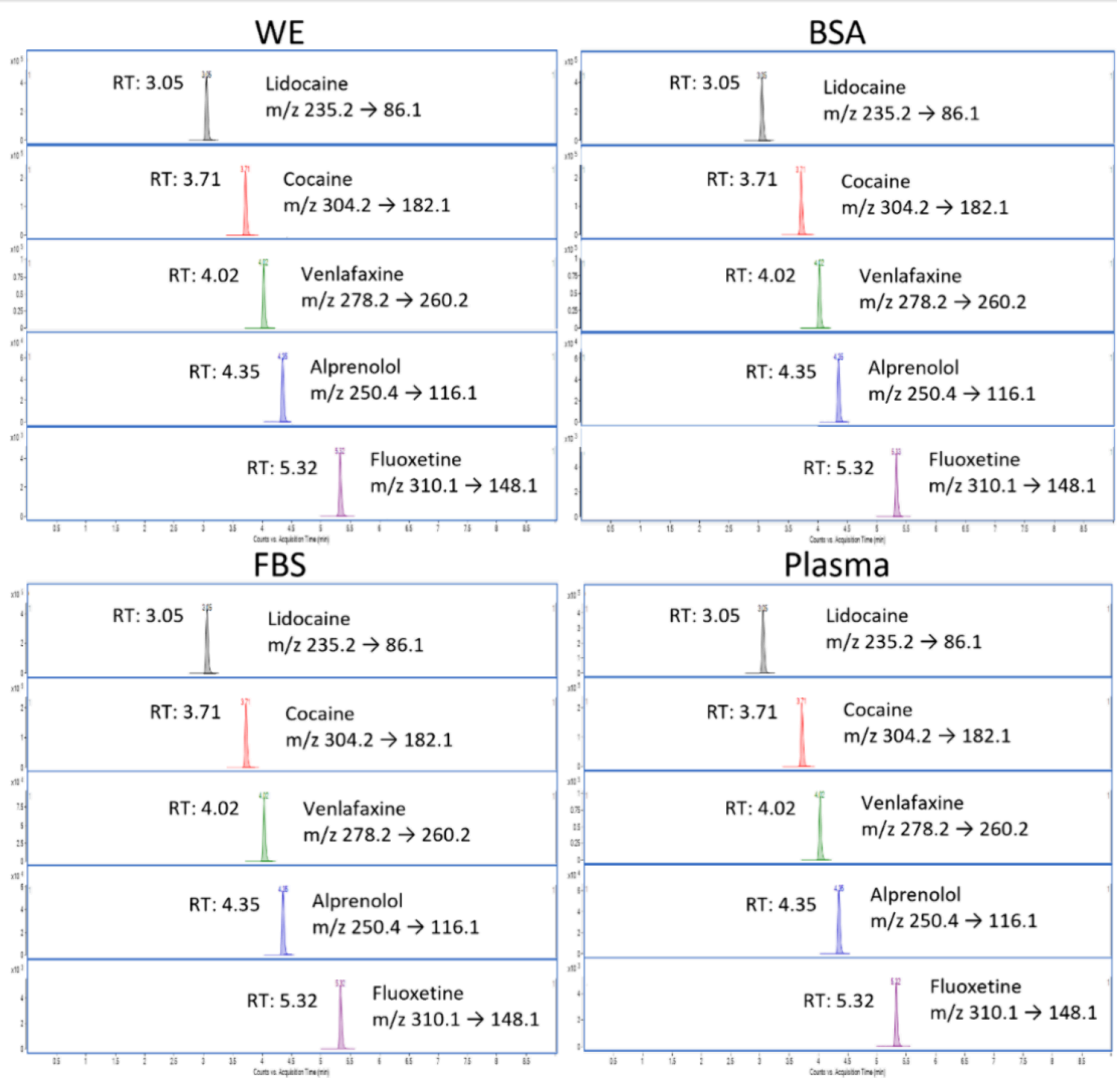
Chromatograms of representative compounds from acceptor solutions for all matrixes showing stable retention times and MS/MS transitions.

The chromatographic performances of all acceptor solutions were similar, in that the peak shapes and retention times were identical, and no pressure buildup was observed for the samples (representative chromatograms shown in Figure 5).

### Demonstration of the System Using Liver Organoids

**Figure 6:**
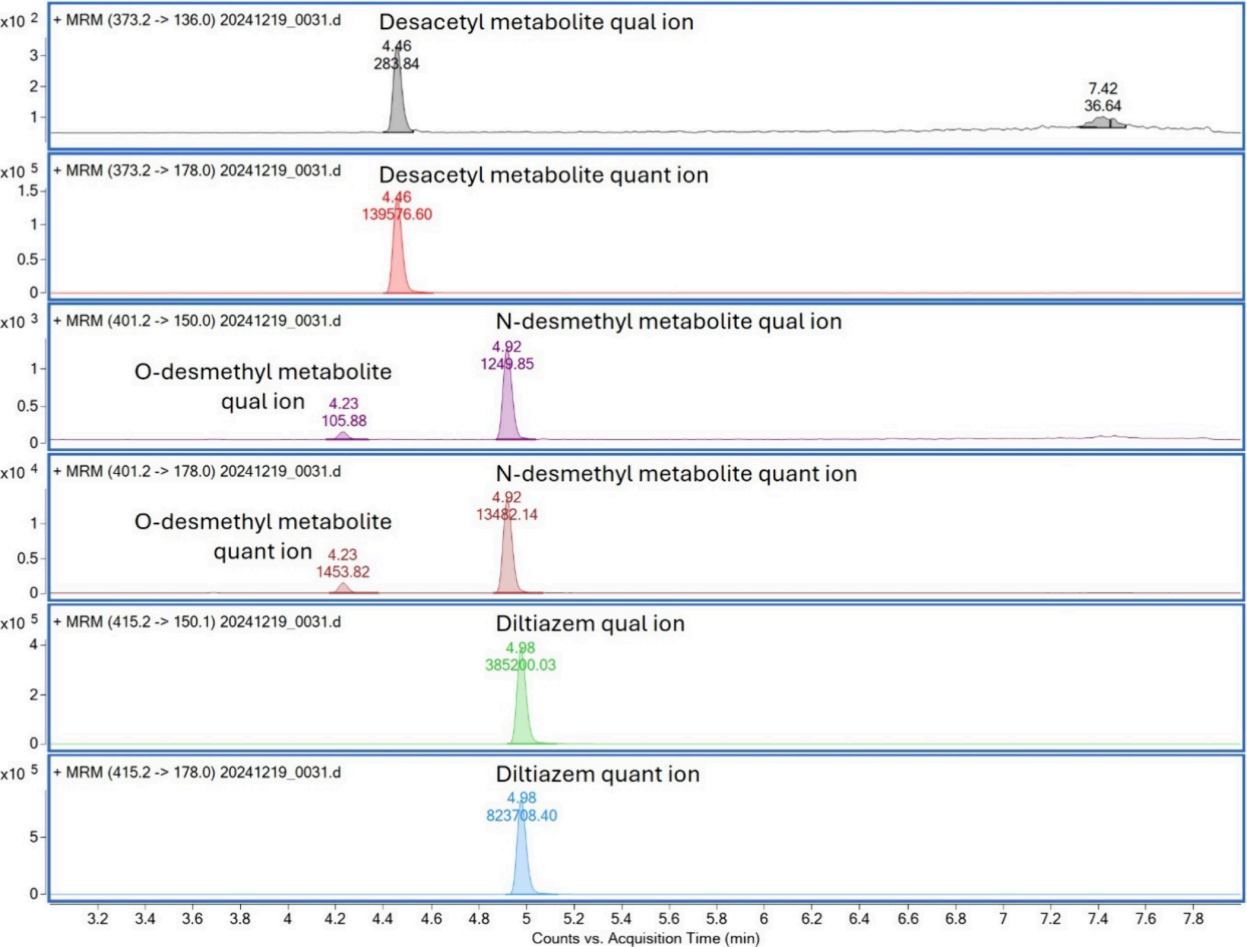
Representative SRM transitions of diltiazem and metabolites when exposed to liver organoids. LC–MS conditions were similar to those of the method for 90 alkaline substances but using MRM mode with a 30 ms dwell time for each transition.

**Figure 7:**
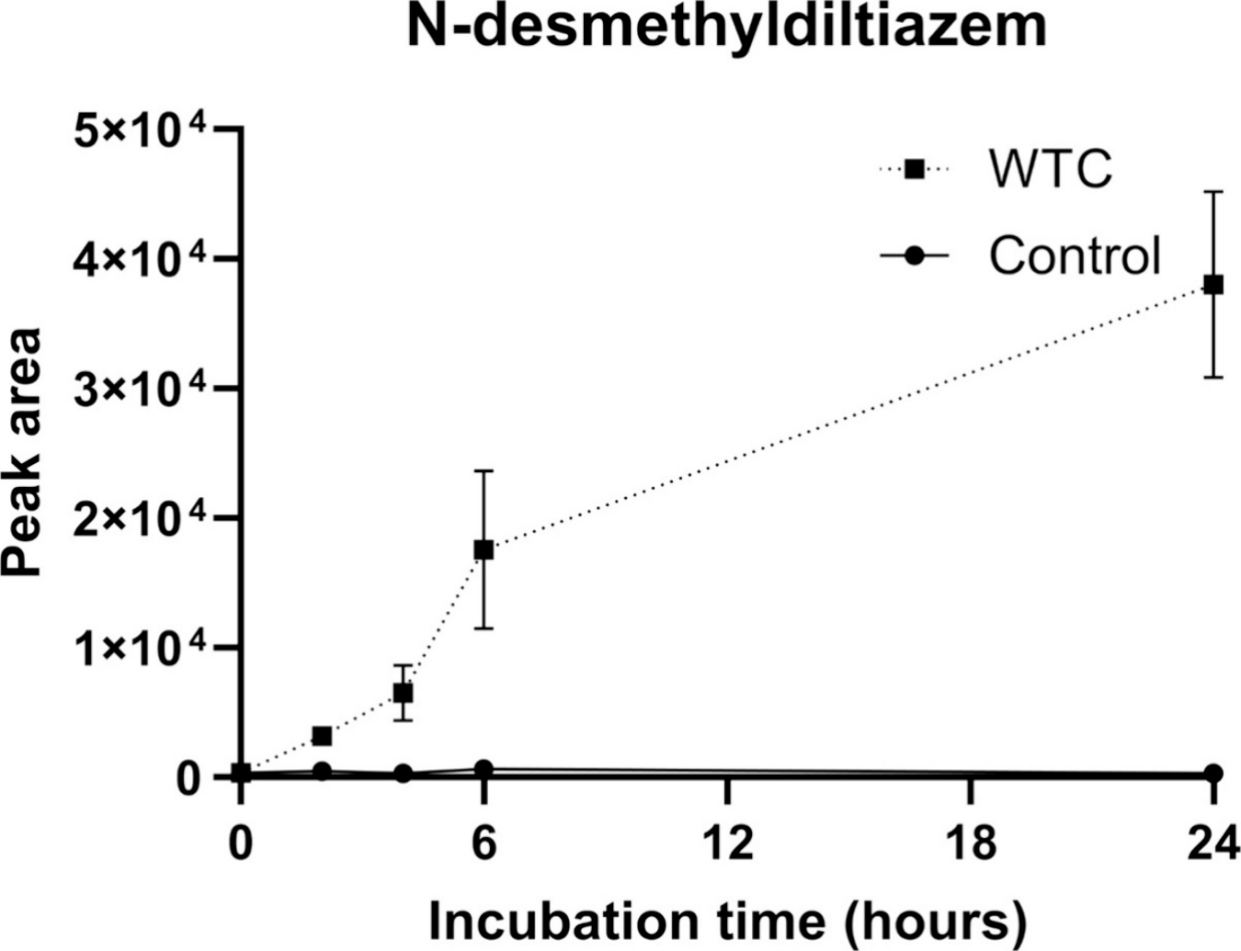
Increase of *N*-desmethyl diltiazem (CYP3A4 metabolism of diltiazem) during 24 h incubation in stem-cell-derived liver organoids. Error bars represent the standard deviation of three biological replicates, each extracted with a single EME replicate.

A key application of the system may be probing cytochrome P450 activity in organoids and related biomaterials, e.g., for quality control and benchmarking of organoids and organoid protocols, for drug development, and for personalized testing. Human liver organoids were incubated with 1 μM diltiazem to scout for telltale metabolites of cytochrome P450 (CYP) enzymes, and samples were taken for measurements at 0, 6, 12, 18, and 24 h. Figure 6 demonstrates the performance of the system on media derived from human liver organoids, showing peaks with quality similar to that of those in the previous experiments. The drug and metabolites deacetyl diltiazem and *N*-desmethyl diltiazem/*O*-desmethyl diltiazem (associated with esterases and CYP3A4 and CYP2D6 metabolism, respectively) were clearly detected and unperturbed using the one-step sample EME sample preparation. *N*-Desmethyl diltiazem levels (representing CYP3A4 metabolism) were significantly higher than *O*-desmethyl diltiazem levels, which represent the (expected) less pronounced CYP2D6 metabolism of the drug. Deacetylation (deacetyl diltiazem) was also clearly observed, but not necessarily associated with enzyme-based activity, as metabolism in controls was also present; we have previously also observed this effect in other drugs with esterase-related degradation.^19^ Focusing on the CYP3A4-associated metabolite *N*-desmethyl diltiazem, a clear metabolism over 24 h was observed (Figure 7).

## Conclusions

Taken together, we observed EME to provide highly selective extractions of drugs compared to potential interferences in the matrixes of emerging biosamples/systems, namely, organoids and OoCs. SDS-PAGE analysis is a convenient tool for assessing cleanup when developing new EME conditions and sample preparations of biosamples in general, visualizing the highly efficient removal of proteins from CCM samples, also compared to more traditional PPT approaches. NMR spectroscopy is useful for assessing an overall cleanup regarding organic molecules and shows that EME is also an excellent approach for removing small molecules in CCM solutions. Using LC–MS, we observed that EME provides extraction windows of drugs for CCM, dependent on log *P* values. The approach allowed for a simple analysis of drug metabolism by liver organoids. The log *P* range of the EME setup does not span the log *P* of small-molecule CCM components, allowing for highly selective analysis, which may allow for simpler detectors for high-throughput screening of known drugs/metabolites in organoid/OoC samples, e.g., in flow-through chip format. However, methods must be validated with a focus on variance that can occur due to the type of CCM and additives (e.g., acid to donor solution or not).

## Supporting Information Available

Table of drugs in the spiking mixture, unedited images of SDS-PAGE gels, and large images of NMR spectra (PDF)
