# Supplementary material for: Electromembrane Extraction Provides Unprecedented Selectivity for Drugs in Cell Culture Media Used in Organoid and Organ-on-Chip Systems
Source: Anal Chem. 2025 Feb 25;97(9):4923–31. doi: 10.1021/acs.analchem.4c04994 (PMC11912128; doi:10.1021/acs.analchem.4c04994)
Supplement: Supplementary file 1 — ac4c04994_si_001.pdf [file ac4c04994_si_001.pdf]

Supporting information for:

Electromembrane extraction provides unprecedented selectivity for drugs in cell culture media used in organoid and organ-on-chip systems

Stian Kogler [1,2], Frøydis Sved Skottvoll [3], Helena Hrušková [1,2], Frode Rise [4], Aleksandra Aizenshtadt[2], Stefan Krauss[2], Hanne Røberg-Larsen [1,2], Frederik André Hansen [5], Steven Ray Wilson* [1,2]

[1] Section for Chemical Life Sciences, Department of Chemistry, Faculty of Mathematics and Natural Sciences, University of Oslo, 0371 Oslo, Norway

[2] Hybrid Technology Hub - Centre of Excellence, Institute of Basic Medical Sciences, Faculty of Medicine, University of Oslo, 0315 Oslo, Norway

[3] Department of Smart Sensors and Microsystems, SINTEF Digital, 0373 Oslo, Norway

[4] Section for Catalysis and Organic Chemistry, Department of Chemistry, Faculty of Mathematics and Natural Sciences, University of Oslo, 0371 Oslo, Norway

[5] Department of Pharmacy, Faculty of Mathematics and Natural Sciences, University of Oslo, 0371 Oslo, Norway

* Steven Ray Wilson, Section for Chemical Life Sciences, Department of Chemistry, Faculty of Mathematics and Natural Sciences, University of Oslo, 0371 Oslo, Norway. Telephone: +47 97010953. E-mail: stevenw@kjemi.uio.no

Table of content:

Table of drugs in spiking mixture……………………………………………………………..S2

Unedited images of SDS-PAGE gels…………………………………………………………..S4

Large image overlays of NMR spectra of acceptor and donor…………………..S6

Supporting information 1 – Overview over drugs included in the spiking mixture, including their mass, strongest acidic and basic pKa and log P values.

Name	Molar mass	Acidic pKa	Basic pKa	logP
**2,6-di-tert-butyl-4-(dimethylaminomethyl)phenol**	263,425	10,75	8,8	4,602
**6-MAM**	327,38	10,19	9,08	1,307
**Acetyl choline**	146,209			-4,221
**Adenine**	135,13	10,29	3,66	-0,531
**Alprenolol**	249,354	14,09	9,67	2,693
**Amantidine**	151,253		10,71	1,466
**Amiodarone**	645,32		9,08	7,635
**Amitriptyline**	277,411		9,76	4,81
**Antipyrin**	188,23		0,49	1,219
**Atenolol**	266,341	14,08	9,67	0,425
**Atropine**	289,375	9,39	15,15	1,571
**Benzamidine**	120,155	11,53		0,894
**Bumetanide**	364,42	3,69	2,7	2,423
**Butylhydrazine**	88,154	NaN	8,49	0,514
**Chlorpromazine**	318,86	NaN	9,2	4,535
**Chlorprothixene**	315,86	NaN	9,76	5,066
**Cimetidine**	252,34	10,13	6,53	-0,109
**Cinnarizine**	368,524	NaN	8,1	5,88
**Clofazimine**	473,4		5,89	7,304
**Clomipramine**	314,86	NaN	9,2	4,883
**Clotrimazole**	344,84		6,26	5,839
**Cocaine**	303,358	NaN	8,85	2,282
**Denatonium**	325,475	NaN		0,405
**Diltiazem**	414,52	8,18	12,86	2,727
**Dopamine**	153,181	9,27	10,01	0,03
**Doxepin**	279,383	9,76		3,84
**Droperidol**	379,435	6,75	12,72	3,014
**Enalapril**	376,453	5,2	3,67	0,588
**Ephedrine**	165,236	9,52	13,89	1,318
**Epinephrine**	183,207	8,91	9,69	-0,43
**Famotidine**	337,44	7,97	10,03	-1,95
**Fluoxetine**	309,332	9,8		4,173
**Halofantrine**	500,43	14,47	10,2	8,057
**Haloperidol**	375,87	13,96	8,2	3,661
**Hydralazine**	160,18		3,9	0,751
**Hydroxyzine**	374,91	15,12	7,45	3,413
**Ipratropium**	332,463	15,15		-1,818
**Isoniazid**	137,142	13,61	3,35	-0,69
**Lidocaine**	234,343	13,78	7,75	2,843
**Loperamide**	477,05	13,96	9,56	4,771

**Luminol**	177,163	8,61	1,61	-0,064
**Meclizine**	390,96		7,71	6,388
**Mepiquat**	114,211			-3,122
**Metaraminol**	167,208	9,03	9,68	-0,045
**Metformin**	129,167	15,14	12,29	-0,918
**Methadone**	309,453		9,12	5,007
**Metoprolol**	267,369	14,09	9,67	1,759
**Mianserin**	264,372		6,92	3,831
**N-acetylputrescine**	130,191		9,9	-1,026
**N-guanylurea**	102,097	13,6	9,79	-2,034
**Nicotinamide**	122,127	13,39	3,63	-0,394
**Nortriptyline**	263,384		10,47	4,426
**Noscapine**	413,426		7,14	2,581
**O-desmethylvenlafaxine**	263,381	10,13	9,01	2,274
**Oxprenolol**	265,353	14,09	9,67	2,168
**Papaverine**	339,391		6,03	3,08
**Perphenazine**	403,97	15,59	7,81	3,692
**Pethidine**	247,338		8,16	2,456
**Pimozide**	461,557	12,9	8,38	5,826
**Piperazine**	86,138		9,56	-0,729
**Practolol**	266,341	14,03	9,67	0,832
**Procaine**	236,315		8,96	1,88
**Prochlorperazine**	373,94		7,99	4,382
**Promazine**	284,42		9,2	3,931
**Promethazine**	284,42		9,05	4,288
**Propranolol**	259,349	14,09	9,67	2,584
**Pyridoxine**	169,18	9,4	5,58	-0,951
**Pyrilamine**	285,391		8,76	3,044
**Quinine**	324,424	13,89	9,05	2,513
**Raloxifene**	473,59	9	8,42	5,465
**Ranitidine**	314,4	7,8		0,991
**Reserpine**	608,688	7,02		3,531
**Salbutamol**	239,315	9,4	10,12	0,344
**Serotonin**	176,219	10	9,31	0,482
**Sotalol**	272,36	9,43	10,07	-0,395
**Sulfadiazine**	250,28	2,01	6,99	0,387
**Sulfamethazine**	278,33	2	6,99	0,65
**Sulfamethoxalol**	253,28	1,97	6,16	0,791
**Tamoxifen**	371,524		8,76	6,351
**Telmisartan**	514,629	3,62	5,86	6,13
**Thiamine**	265,35	5,54	15,5	-3,097
**Thioridazine**	370,57	8,93		5,469
**Timolol**	316,42	9,76	14,08	1,336
**Triclabendazole**	359,65	10,31	4,39	5,884
**Triisopropanolamine**	191,271	9,28	14,81	-0,63

**Trimipramine**	294,442	9,42		4,758
**Tyramine**	137,182	9,66	10,41	0,68
**Tyrosine methyl ester**	195,218	6,99	9,51	0,92
**Venlafaxine**	277,408	9,06	14,42	2,739
**Verapamil**	454,611	9,68		5,043

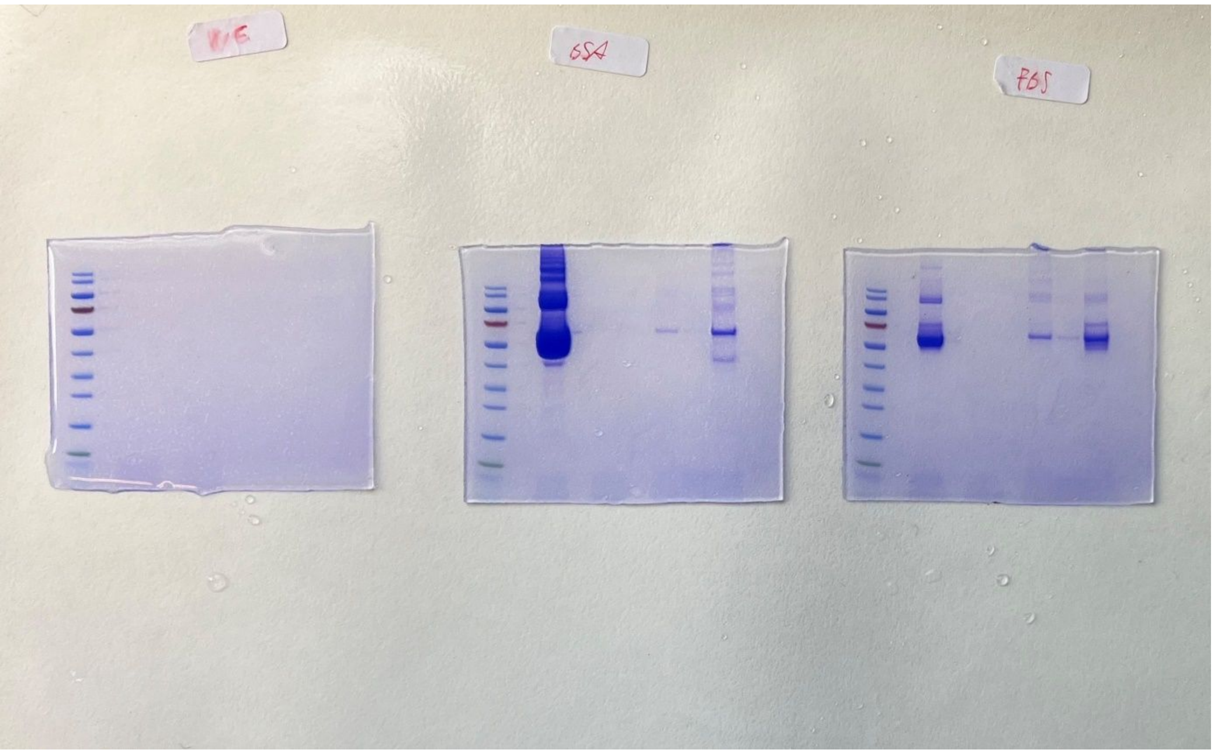

Supporting information 2 – Unedited image of SDS-PAGE gels for WE-medium, BSA-medium and FBS-Medium samples.

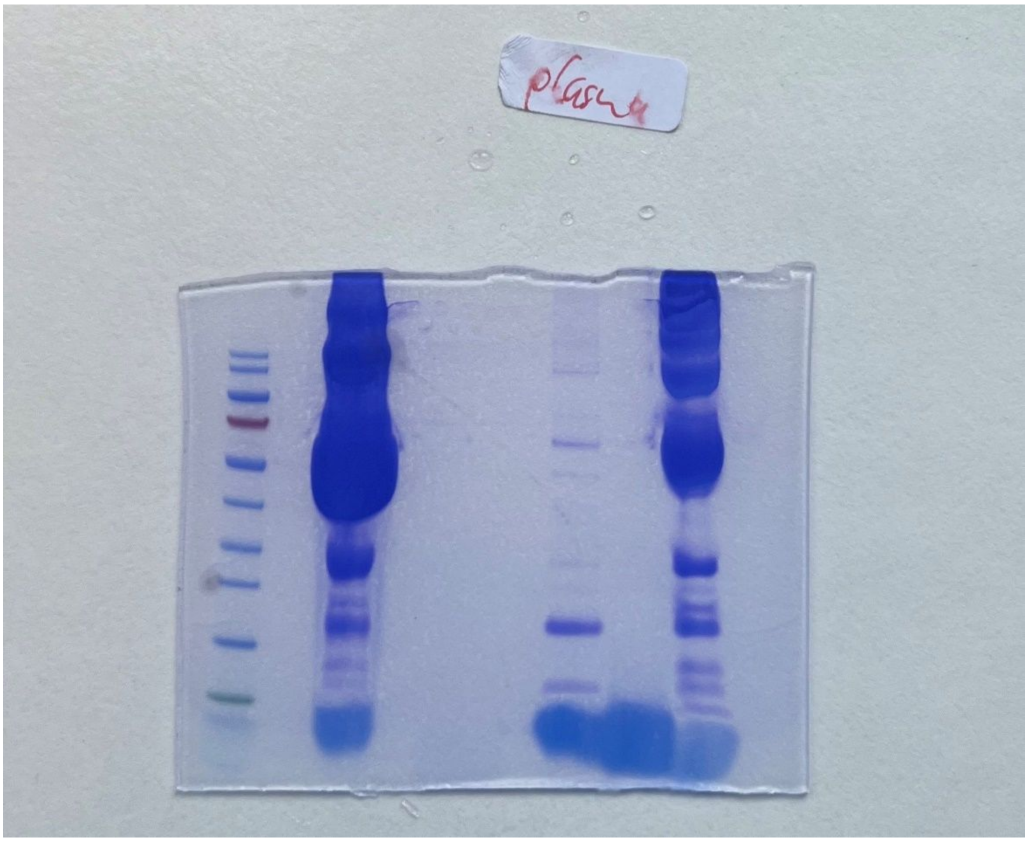

Supporting information 3 – Unedited image of SDS-PAGE gel for human plasma samples

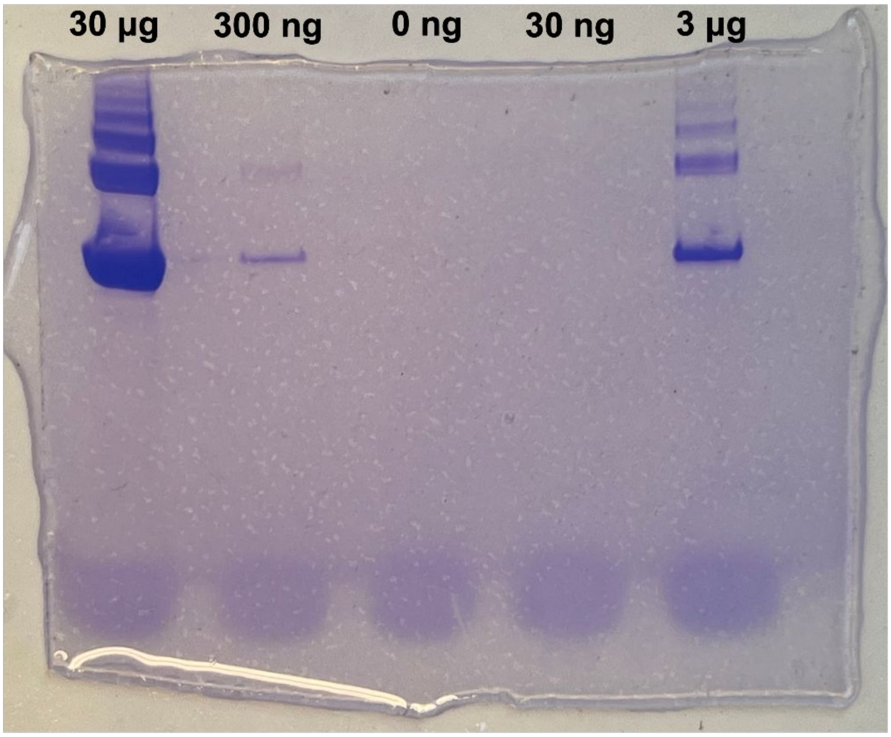

Supporting information 4 – Unedited image of SDS-PAGE gel of dilution series of BSA in the range 0-30 µg for estimation of LOD. The loaded amount of protein is stated in labels above the gel. LOD of 30-300 ng was estimated visually by students not related to the work.

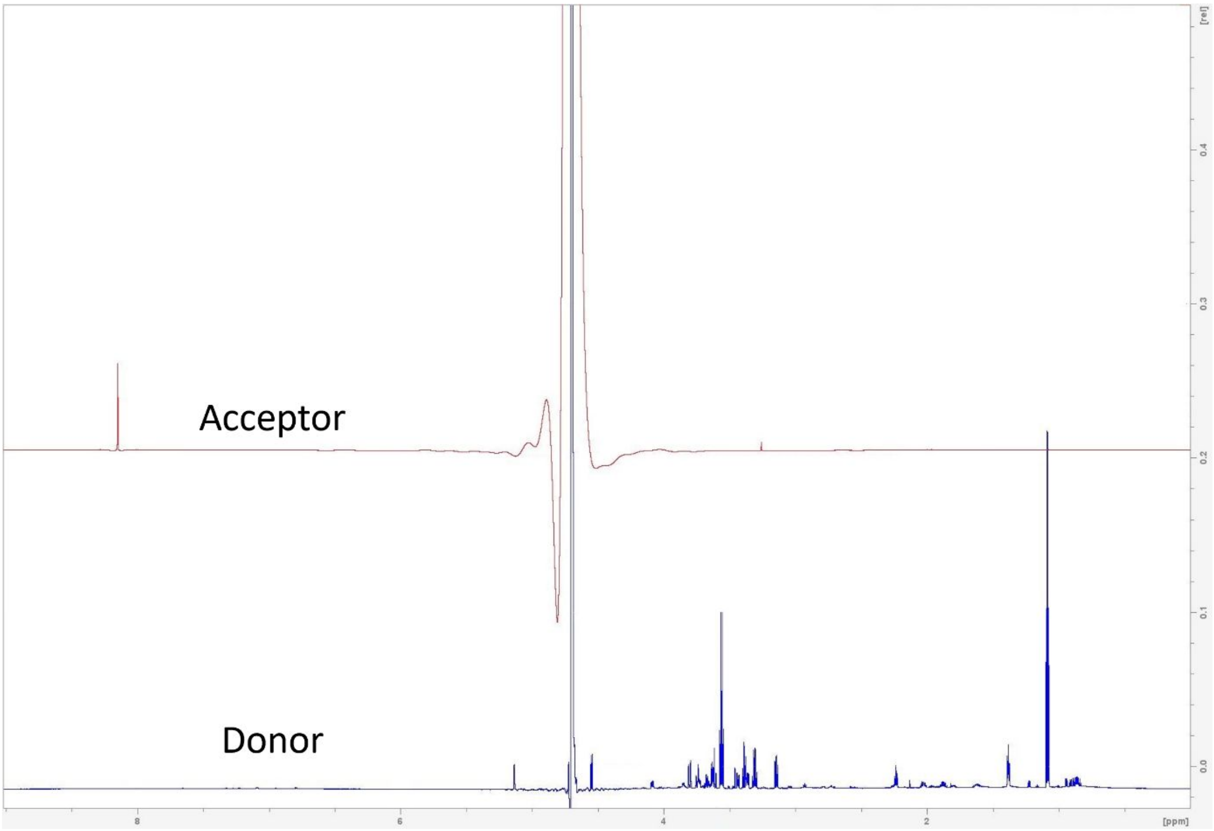

Supporting information 5 - Overlay of NMR spectra of WE medium prior to EME (donor) and of the extract (acceptor) after EME.

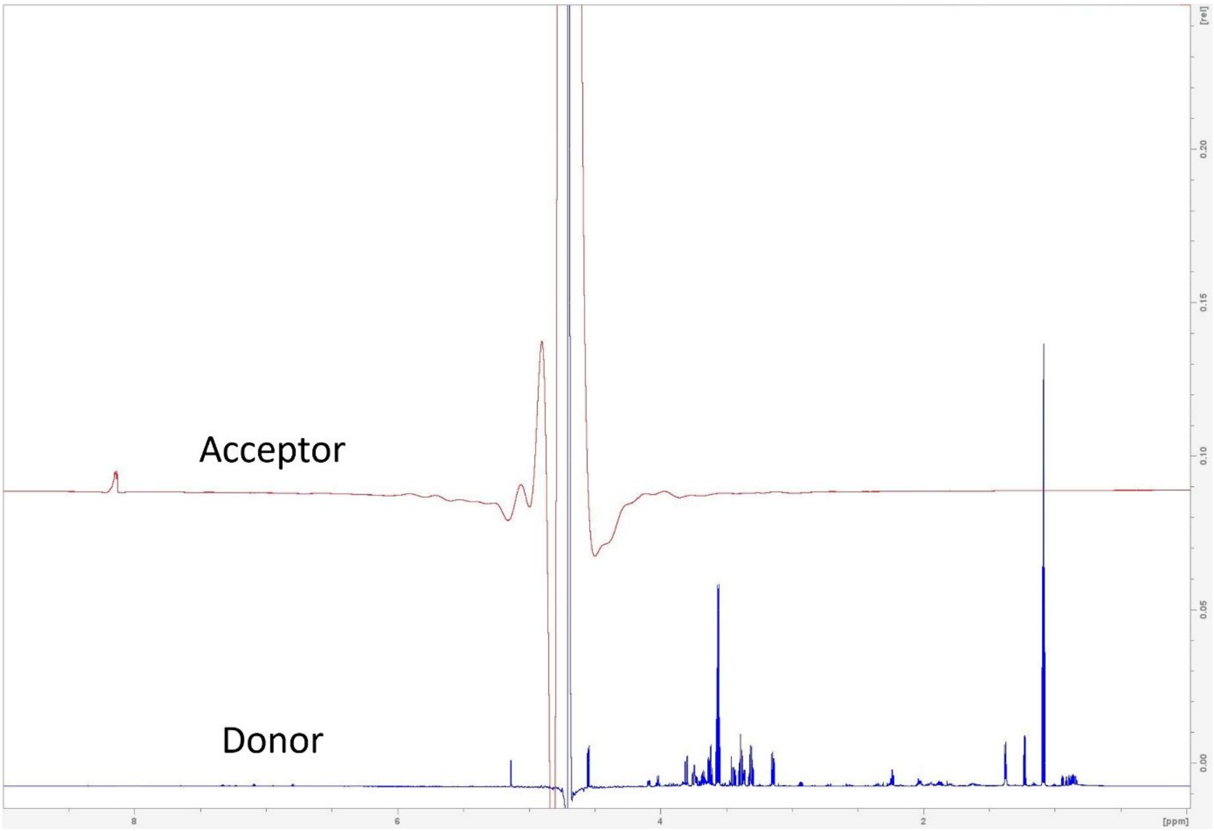

Supporting information 6 - Overlay of NMR spectra of 1% BSA-medium prior to EME (donor) and of the extract (acceptor) after EME.

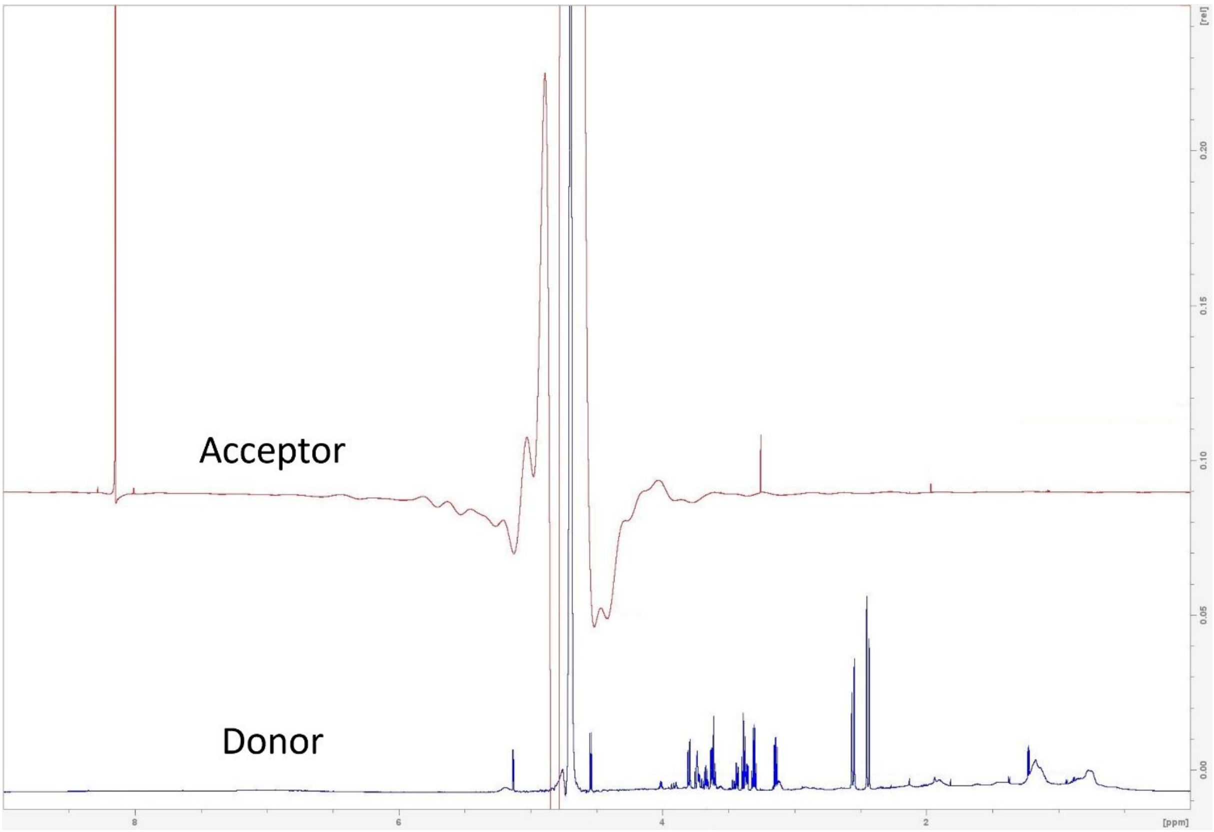

Supporting information 7 - Overlay of NMR spectra of 1%-FBS medium prior to EME (donor) and of the extract (acceptor) after EME.

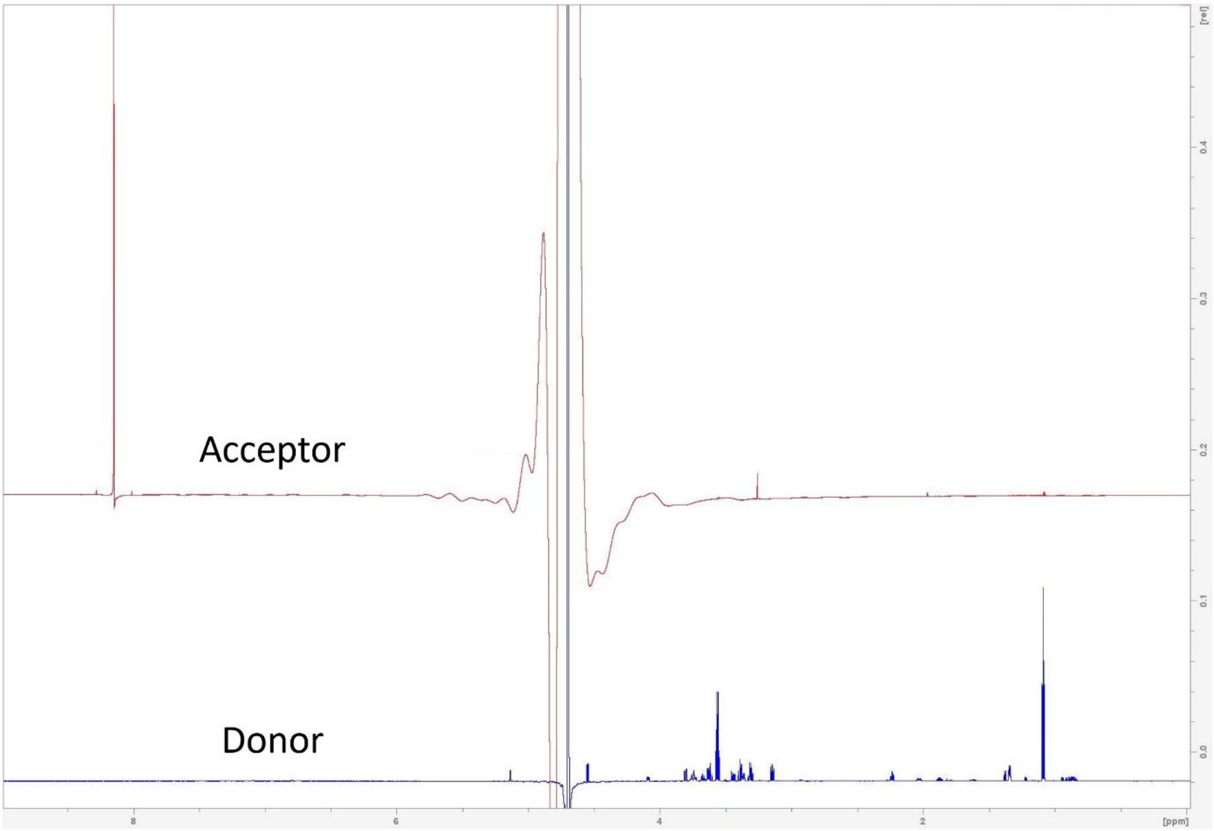

Supporting information 8 - Overlay of NMR spectra of human plasma prior to EME (donor) and of the extract (acceptor) after EME.
